# Opto-thermoelectric speckle tweezers

**DOI:** 10.1515/nanoph-2019-0530

**Published:** 2020-03-07

**Authors:** Abhay Kotnala, Pavana Siddhartha Kollipara, Yuebing Zheng

**Affiliations:** Walker Department of Mechanical Engineering, Materials Science and Engineering Program and Texas Materials Institute, The University of Texas at Austin, Austin, TX 78712, USA; Walker Department of Mechanical Engineering, Materials Science and Engineering Program and Texas Materials Institute, The University of Texas at Austin, Austin, TX 78712, USA

**Keywords:** thermophoresis, optical tweezers, speckle, opto-thermoelectric tweezers

## Abstract

Opto-thermoelectric tweezers present a new paradigm for optical trapping and manipulation of particles using low-power and simple optics. New real-life applications of opto-thermoelectric tweezers in areas such as biophysics, microfluidics, and nanomanufacturing will require them to have large-scale and high-throughput manipulation capabilities in complex environments. Here, we present opto-thermoelectric speckle tweezers, which use speckle field consisting of many randomly distributed thermal hotspots that arise from an optical speckle pattern to trap multiple particles over large areas. By further integrating the speckle tweezers with a microfluidic system, we experimentally demonstrate their application for size-based nanoparticle filtration. With their low-power operation, simplicity, and versatility, opto-thermoelectric speckle tweezers will broaden the applications of optical manipulation techniques.

## 1 Introduction

Nanoparticle manipulation tools have become important for scientific research and engineering applications in numerous fields ranging from biophysics to nanomanufacturing [1–10]. New tweezing platforms with improved capabilities are being developed to meet the ever-expanding applications [11–20]. The recently invented opto-thermoelectric tweezers have emerged as an alternative for optical trapping and manipulation of nanoparticles [21, 22]. Based on the electrolyte Seebeck effect [23], opto-thermoelectric tweezers can manipulate particles of variable sizes, shapes, and material compositions with low-power optics and simple instrumentation [21, 24]. Their applications in nanoparticle assembly [25–27], printing [28, 29], and delivery [30] have been demonstrated.

Like conventional optical tweezers, opto-thermoelectric tweezers have limited applications because of low throughput in trapping and manipulating individual particles. In an optical tweezers system, patterning laser beams using holographic diffractive elements [31, 32] and standing waves in free space [33, 34] or waveguides [35] has enabled large-scale and high-throughput trapping of particles over extended areas. Although such techniques can be extended to opto-thermoelectric tweezers, the use of sophisticated optical components or advanced fabrication tools will limit their practical applications. Alternatively, random light fields known as “speckles”, which are generated from the interference of dephased but coherent monochromatic light waves, have been proposed for trapping and manipulation of particles [36]. One of the earliest demonstrations showed trapping and cooling of caesium atoms at the high-intensity spots of the speckle field [37]. Ingeniously, dark regions within a volumetric speckle field have also been exploited to trap thousands of airborne absorbing particles based on the photophoretic forces [38, 39]. Speckle tweezers based on optical gradient force were also proposed theoretically [40] and later demonstrated experimentally along with applications in the study of anomalous diffusion in colloids and sorting of particles [41, 42].

Here, we present opto-thermoelectric speckle tweezers (OTEST) based on thermal speckle field and their application for large-scale and high-throughput manipulation of nanoparticles. A thermal speckle field consisting of many thermal hotspots, which can trap a large number of particles using thermoelectric forces, was generated by merging an optical speckle field with a plasmonic substrate. The plasmonic substrate enhanced the opto-thermal conversion. OTEST were able to optically trap both metallic and dielectric particles as small as 100 nm in radius using low optical power [42]. Our OTEST could be readily integrated into microfluidic systems for advanced particle manipulations. By controlling the balance between the thermoelectric force and drag force from localized convection flow acting on the particles in OTEST, we achieved high-throughput optical filtration of nanoparticles based on their sizes. We envision that speckle-based opto-thermoelectric tweezers will expand the functionalities and applications of light-based manipulation techniques.

## 2 Results and discussion

### 2.1 Experimental setup and particle-trapping mechanism

**Figure 1:**
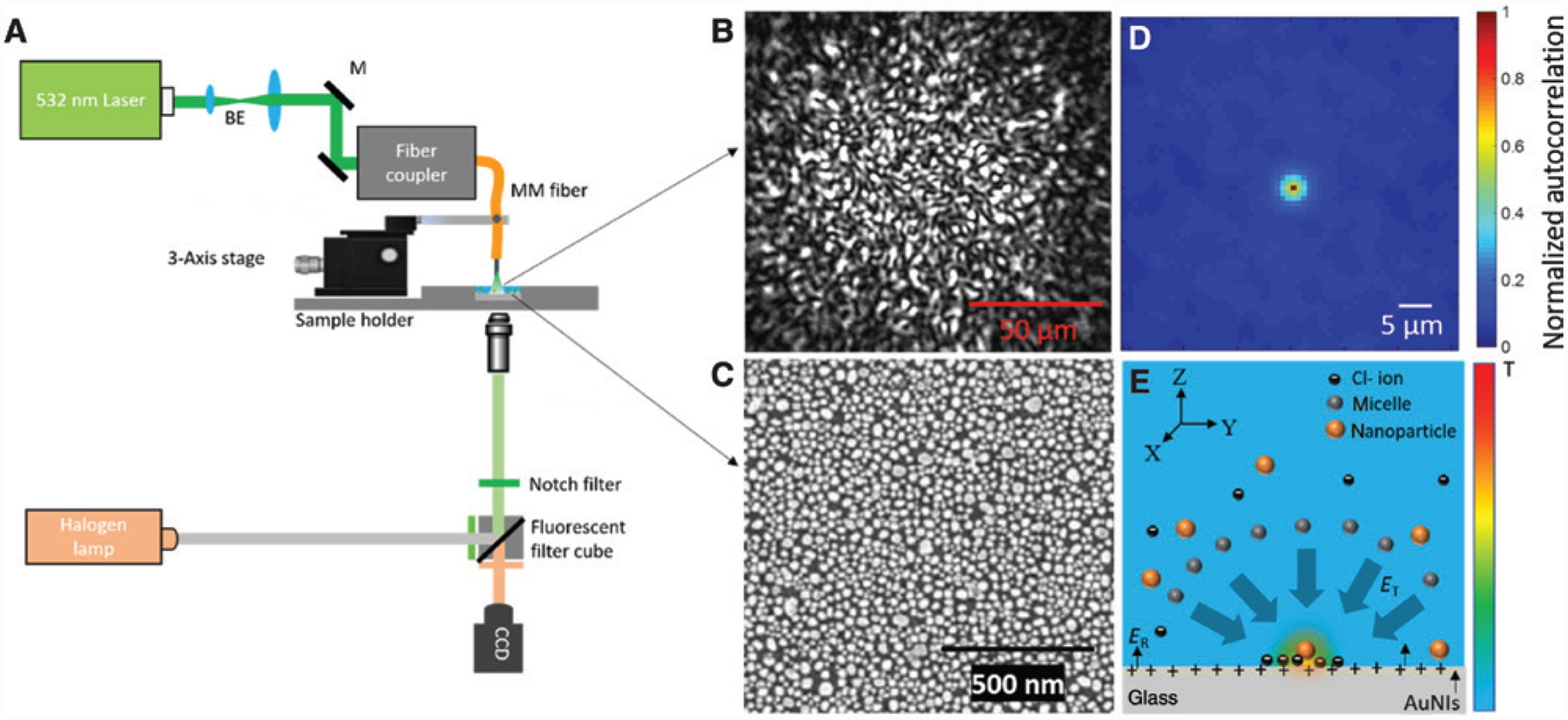
Opto-thermoelectric speckle tweezers. (A) Schematic of the experimental setup of opto-thermoelectric speckle tweezers (BE: beam expander, M: mirror, CCD: charge-coupled device). (B) Optical image of a typical speckle light pattern generated at the output of an MM fiber with a diameter of 105 μm. (C) Scanning electron microscope image of a plasmonic AuNIs substrate. (D) Normalized spatial autocorrelation function of the speckle pattern shown in B to estimate the average speckle grain size. (E) Schematic of the nanoparticle trapping mechanism of opto-thermoelectric speckle tweezers. The solution consists of micellar ions, counter-ions, and the nanoparticles to be trapped. Embedded in the schematic is the temperature gradient (also see color bar at the right side) induced by a hotspot in the thermal speckle field.

A schematic of the experimental setup of an OTEST is shown in Figure 1A. A thermal speckle field consisting of many randomly distributed thermal hotspots used for trapping of nanoparticles was generated by directing the output of a multimode (MM) fiber (core diameter = 105 μm, numerical aperture = 0.22) on a plasmonic gold nano-island (AuNI) substrate (Figure 1C). The fiber was coupled to a laser (Genesis MX STM-1 W; Coherent λ = 532 nm). The output of the MM fiber was a speckle light pattern (Figure 1B) that arose from the strong interference among a large number of modes excited in the MM fiber [43, 44]. The optical speckle field led to a thermal speckle field when it was incident on the plasmonic substrate, which converted the high-intensity optical speckle grains into corresponding thermal speckle grains or hotspots due to the high opto-thermal conversion efficiency of AuNIs [15]. The average hotspot size in the optical speckle field was estimated to be around 2 μm by calculating the full width at half-maximum of the normalized spatial autocorrelation function of the speckle pattern as shown in Figure 1D [43]. Alternative methods such as scattering of a laser beam from a surface with high roughness like a diffuser [45] and transmission of light through a complex scattering medium can also be used to generate optical speckle patterns. However, speckle patterns generated from an MM fiber have advantages of uniform speckle distribution, easy alignment with the AuNI substrate, high optical transmission efficiency, and high flexibility to expand the thermal speckle field by simply using multiple fibers.

As shown in Figure 1E, to generate thermoelectric forces that were responsible for the trapping of nanoparticles in OTEST, a cationic surfactant, cetyltrimethylammonium chloride (CTAC), was added to the solution. Above the critical micelle concentration (0.13–0.16 mm), CTAC formed micelles of nanoscale size with high charge density, which acted as macro-cations (known as micellar ions), whose concentration *c*_mic_ is given by

$$c_{\text{mic}}=\frac{\left(n_{0}-n_{\text{cmc}}\right)}{N_{\text{agg}}}$$

where *N*_agg_ is the aggregation number (~89 for CTAC [46]), *n*_0_ is the concentration of CTAC (2 mm in our experiments), and *n*_cmc_ is the critical micelle concentration of CTAC (~0.13 mm). The Cl^−^ ions act as counter-ions with concentration similar to the CTAC concentration in the solution (each CTAC molecule consist of a single Cl^−^ ion). The temperature gradient generated at each speckle hotspot at the substrate (see Supplementary Information: Section 1) induced the thermophoresis of the micellar ions and Cl^−^ ions [47]. Because of the high molecular mass and large Soret coefficient of CTAC micelles compared to Cl^−^ ions, i.e. *S*_T_ (micelle) ~10^−2^ K^−1^ > *S*_T_ (Cl^−^) ~7.18 × 10^−4^ K^−1^, there was a spatial redistribution of the CTAC micelles and Cl^−^ ions that were otherwise uniformly distributed in the solution. The spatial redistribution of CTAC and Cl^−^ ions generates an electric field *E*_T_ pointing towards the hotspot, as shown in Figure 1E, which is given by [48]

$$E_{\text{T}}=\frac{k_{B}T\nabla T}{e}\frac{\underset{i}{\sum }Z_{i}n_{i}S_{Ti}}{\underset{i}{\sum }Z_{i}^{2}n_{i}}$$

where *i* indicates the ionic species (CTAC micellar ions or Cl^−^ ions), *k*_B_ is the Boltzmann constant, *T* is the solution temperature, ∇*T* is the temperature gradient, *e* is the elemental charge, and *Z*_*i*_, *n*_*i*_, and *S*_T*i*_ are the charge number, the concentration, and the Soret coefficient of *i* species, respectively. The positively charged nanoparticles (due to the formation of a positively charged molecular double layer by CTAC molecules adsorbed on the particle surface [49]) experience a trapping force given by *F*_T_ = *qE*_T_ (*q* is charge on the particles) and got trapped at the thermal hotspot. The trapping electric field (*E*_T_) is balanced by the repulsive field, *E*_R_, which arose from the positively charged substrate coated by the CTAC double layers, as shown in Figure 1E. Unlike the gradient forces in optical tweezers, the thermoelectric forces do not directly depend on the size or material properties of the particles being trapped, indicating that OTEST is versatile in trapping and manipulating various particles with unfocused low-power laser beams.

### 2.2 Large-scale trapping of nanoparticles

**Figure 2:**
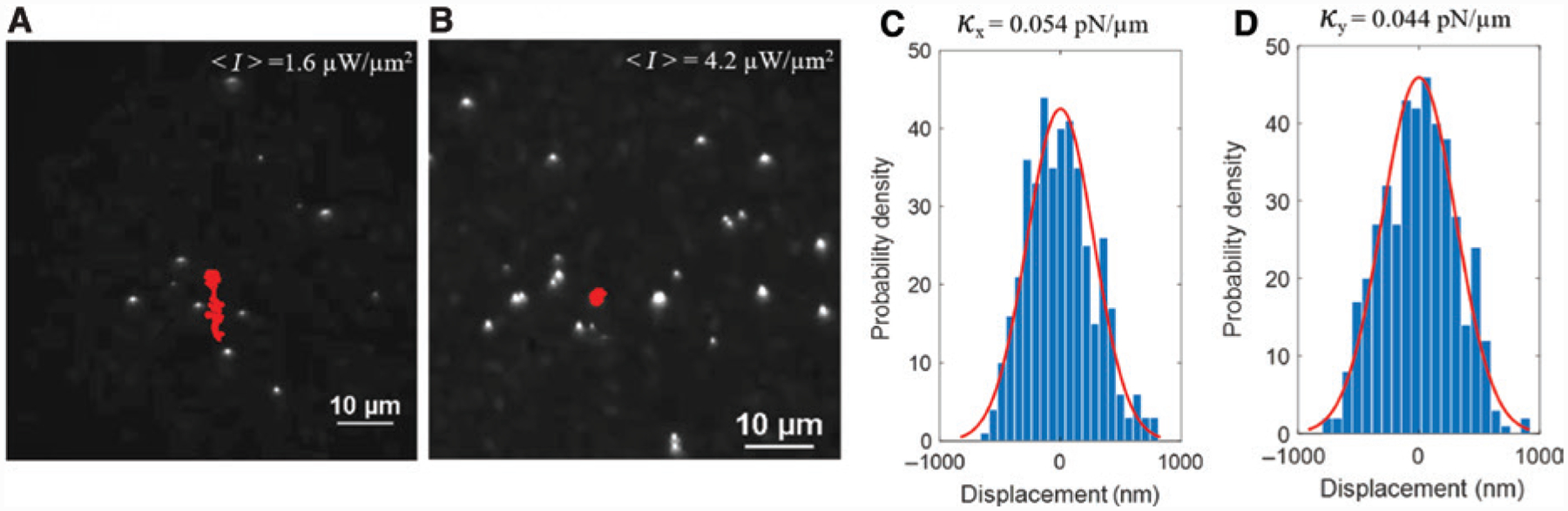
Large-scale trapping of particles with opto-thermoelectric speckle tweezers. Trapping of multiple 500-nm polystyrene particles in a thermal speckle field at an average speckle intensity of (A) <*I*> = 1.6 μW/μm^2^ and (B) <*I*> = 4.2 μW/μm^2^. The red solid lines show the trajectory of one of the particles trapped in the speckle field. (C, D) Positional fluctuations in the *X*- and *Y*-direction (see Figure 1E) of a single 500-nm particle trapped at one of the speckle grain of Figure 1B. The red curves are Gaussian fits.

To demonstrate large-scale trapping of nanoparticles using a thermal speckle field, we brought the output end of the MM fiber to the vicinity of an AuNI substrate covered by the particle solution using a three-axis manipulation stage (Nanomax 300, Thorlabs). The trapping process was imaged using a 40× objective lens and a charge-coupled device (CCD; Nikon) camera. Once the speckle field was turned on, multiple nanoparticles were trapped at the various thermal hotspots in the field. The trapping behavior of individual particles varied depending on the average speckle field intensities. For example, at low average speckle intensity of <*I*> = 1.6 μW/μm^2^, the 500-nm polystyrene particles were trapped for short durations in the speckle hotspots and jumped from one hotspot to the next over time, exhibiting a sub-diffusive behavior as evident from the trajectory of one of the particles (Figure 2A). The sub-diffusive behavior is due to the unstable trapping of particles at the hotspots because of the weak thermoelectric force acting on the particles at low speckle intensities. However, at the higher average speckle intensity, i.e. <*I*> = 4.2 μW/μm^2^, the particles remained stably trapped in the speckle hotspots for long durations, as shown in Figure 2B, due to the increased thermoelectric forces on the particle.

The trapping behavior of the nanoparticles in a thermal speckle field depends on random thermal force (Brownian motion) and thermoelectric force acting on the particles. At high speckle intensities, the thermoelectric force acting on the particle is much stronger than the thermal force, leading to the strong localization of the particle in a single speckle hotspot. It should be noted that, for the speckle intensities and size of particles used in the present experiments, the optical gradient and scattering forces were too weak to contribute to the trapping of the particles (see Supplementary Information: Section 2). The negligible effect of the optical forces on trapping was verified by our control experiments, where no trapping of particles occurred in the absence of CTAC in the particle solution. In addition to the large-scale trapping, dynamic manipulation of a large number of nanoparticles was achieved by moving the speckle pattern over the AuNI substrate.

OTEST can trap and manipulate particles of variable sizes and material compositions. As demonstrations, we further achieved the trapping of 1-μm, 2-μm, and 200-nm polystyrene particles, and 200-nm silver nanoparticles in the thermal speckle field (see Supplementary Information: Section 3). To characterize the trapping stiffness of our OTEST, we measured the positional fluctuations of single particles trapped at the thermal speckle grains. Figure 2C,D shows the distributions of the position fluctuations in the *X*- and *Y*-direction (see Figure 1E) of a single 500-nm polystyrene nanoparticle trapped in a thermal speckle grain at an average speckle intensity of 4.2 μW/μm^2^. By fitting the distributions with Gaussian functions, we obtained the variance in the position of the trapped particle and calculated the trapping stiffness as

$$\kappa_{x,y}=\frac{KT}{\sigma_{x,y}^{2}}$$

where κ_*x*,*y*_ is the trapping stiffness in the *X*- and *Y*-direction, σ_*x*,*y*_ is the variance in the position of the particle along the *X*- and *Y*-directions, *K* is the Boltzmann constant, and *T* is the temperature. The trapping stiffness was calculated for 500-nm particles trapped at different hotspots (sampling number *N* = 10) in the thermal speckle field. The average trapping stiffness of OTEST was found to be 0.06 ± 0.05 pN/μm. The large variance in the trap stiffness arises from the variation in the size and intensity of different hotspots in the random thermal speckle field.

### 2.3 Large-scale filtration of nanoparticles

Thermoelectric force is the dominant force responsible for trapping of particles in an opto-thermoelectric tweezers (OTET) [21]. AuNI plasmonic substrates used by OTET require very low laser power, which is typically less than 1 mW, to create a strong temperature gradient and the resultant thermoelectric force that is 2–3 orders of magnitude larger than the optical forces [50]. The magnitude of the thermoelectric forces (which is proportional to the temperature gradient) can be enhanced by increasing the incident laser power, which raises both the temperature and the temperature gradient at the AuNI substrate. However, in addition to the increase in the thermoelectric force acting on the particles, the rise in temperature also generates a localized convection flow at the thermal hotspots, which can induce a drag force on the trapped particles in a direction opposite to that of the thermoelectric force. Thus, at high laser intensities, the trapping behavior of particles in OTET is determined by the magnitude of the two opposing forces, i.e. the thermoelectric force, and the drag force generated from the localized convection flow. By properly adjusting the laser intensity, the balance between the thermoelectric force and the drag force acting on the particles can be controlled to selectively trap particles in thermoelectric tweezers (see Supporting Information: Section 4).

Based on the principle of selective trapping of particles in OTEST, we demonstrate large-scale filtration of 200-nm particles from a mixed solution of 200-nm and 1-μm polystyrene particles. The filtration process is based on the difference in the magnitude of the drag force acting on the particles of different sizes. To filter 200-nm particles from a mixed solution of 200-nm and 1-μm particles, we tune the average speckle field intensity such that the drag force from the localized convection flow is larger than the thermoelectric force acting on the 1-μm particles but smaller than the thermoelectric force on the 200-nm particles. This results in the dislodging of the larger particles from the thermal hotspots, while the smaller particles remain trapped in the thermal speckle field and get filtered out.

**Figure 3:**
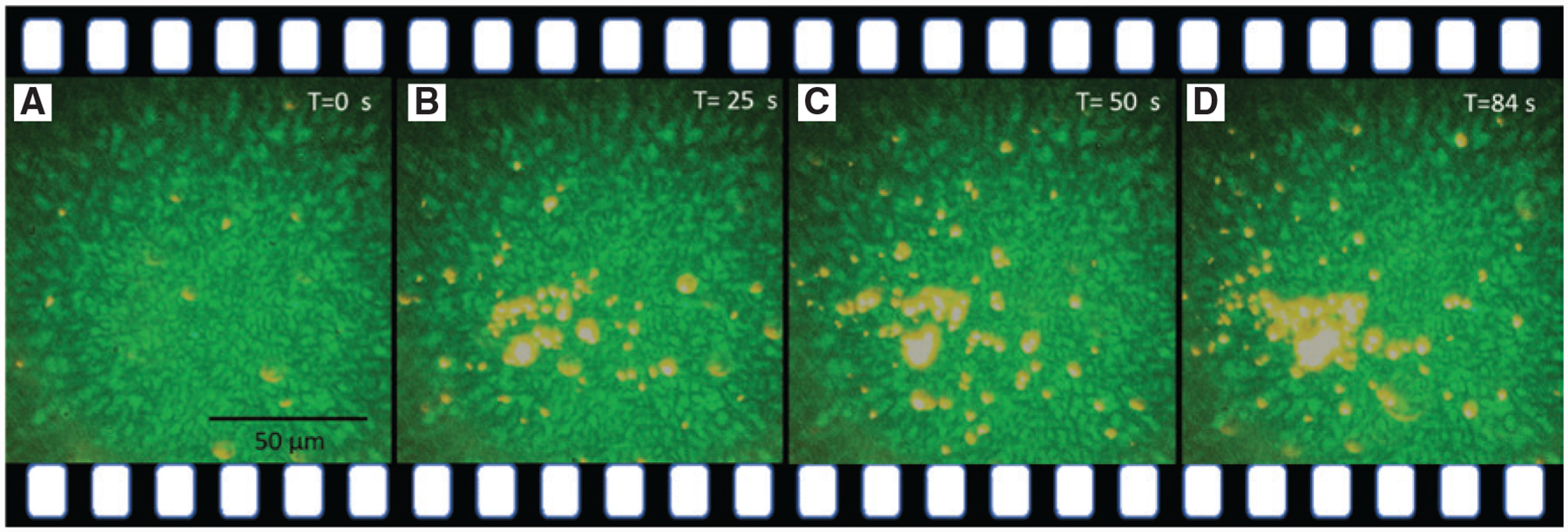
Size-based filtration of nanoparticles using opto-thermoelectric speckle tweezers. (A–D) Time-lapsed images showing that a thermal speckle field could selectively trap a large number of 200-nm nanoparticles from the mixed solution of 200-nm and 1-μm polystyrene particles flowed in an AuNI-embedded microfluidic channel, which enabled large-scale separation or filtration of nanoparticles based on sizes.

To implement particle filtration, we integrated the OTEST with an AuNI-embedded microfluidic channel (see Section 4 and Supplementary Information: Section 5). A mixed solution consisting of 200-nm and 1-μm particles was flown in a AuNI-embedded microfluidic channel with an average flow velocity of 20 μm/s. When the thermal speckle field with an average intensity of <*I*> = 5.2 μW/μm^2^ was turned on, the 200-nm fluorescent nanoparticles accumulated in the speckle field and got filtered out, while the 1-μm particles were carried away by the flow in the channel as shown in Figure 3 (see Supplementary Video 4). At the given speckle intensity, the drag force due to the localized convection flow becomes stronger than the thermoelectric force acting on the 1-μm particles, which results in no trapping of 1-μm particles in the thermal speckle field and being driven away by the fluid flow. In addition, the drag force from the laminar flow in the microfluidic channel also makes it difficult to capture larger particles flowing far away from the speckle hotspots, and get carried away by the flow. In contrast, the thermoelectric force on the smaller 200-nm particles dominates over the drag forces from localized convection flow and laminar flow in the channel. The drag forces acting on the 200-nm particles are smaller compared to those on the 1-μm particles (drag force is directly proportional to size of particle). Therefore, the smaller particles get trapped in the thermal speckle field and are filtered out. This filtration process is unique as it retains the smaller particles while allowing the larger ones to flow through the microfluidic channel, which is opposite to the filtration based on the optical gradient force in optical speckle tweezers [42]. One can further improve the particle filtration efficiency and resolution by better control of the flow velocity, the CTAC concentration, and the thermal speckle field. Once fully developed, our OTEST-based filtration system may be used as an alternative to the ultracentrifuge, thus preventing damage and aggregation of nanoparticles being filtered out of complex fluids.

## 3 Conclusion

OTEST for large-scale and high-throughput trapping and manipulation of nanoparticles were developed. The tweezers exploit thermoelectric forces that arise from the thermal speckle field to trap a large number of particles of variable sizes and material compositions using low-power, unfocused light. By further integrating OTEST with microfluidic systems, we achieved the large-scale optical filtration of nanoparticles based on their sizes. As a simple, cost-effective, versatile, and robust platform, OTEST will find applications in various fields.

## 4 Methods

### 4.1 Fabrication of plasmonic substrates integrated with microfluidic channels

To prepare the plasmonic substrate, AuNIs were fabricated by depositing a Au thin film of thickness 4.5 nm (Denton thermal evaporator, Denton Vacuum, Moores-town, NJ, USA, base pressure: 9 × 10^−6^ torr, deposition rate: 0.1 Å/s) on a glass coverslip, followed by thermal annealing at 550°C in air for 2 h using a box furnace (Thermo Scientific™ Lindberg/Blue M™ Box Furnace, Waltham, MA, USA). SU-8 based microfluidic channels were fabricated on the plasmonic substrate using standard photolithography. Briefly, SU-8 (2005) epoxy-based negative photoresist (Kayaku Advanced Materials, Inc., Westborough, MA, USA) was spin-coated on a plasmonic substrate at the rate of 4000 rpm for 45 s to form a uniform layer of SU-8 with a thickness of 5 μm. A well-designed microfluidic channel was created in the SU-8 layer by following a standard lithography recipe for the photoresist. The microfluidic channel had a width of 300 μm and a depth of 5 μm, equivalent to the thickness of the SU-8 layer. The channel bottom consisted of a layer of AuNIs. The microfluidic channel was sealed with a second glass coverslip with drilled holes as microfluidic inlets and outlets using adhesive bonding. A complete stepwise fabrication process along with the microscopic image of an AuNI-embedded microfluidic channel is provided in Section 5 of the Supplementary Information.

### 4.2 Materials

Fluorescent-labeled 200-nm (excitation/emission: 540/600 nm), fluorescent-labeled 500-nm (excitation/emission: 480/520 nm), and 1-μm polystyrene nanoparticle solutions were prepared by diluting the as-purchased solutions from Bang Laboratories, Fishers, IN, USA in 2 mm CTAC with a final concentration of 1 × 10^7^ particles/ml. A solution of 200-nm silver nanoparticles was prepared by diluting the as-purchased solution (NanoPartz Inc., Salt Lake City, UT, USA) in 10 mm CTAC with a final concentration of 1 × 10^7^ particles/ml.

## Supplementary Material

supplementary notes and vides Supplementary Video 1: Real-time video showing the trapping of both 500-nm (fluorescent-labeled) and 1-μm polystyrene particles at low laser intensity using opto-thermoelectric tweezers.
Supplementary Video 2: Real-time video showing the selective trapping of 500-nm (fluorescent-labeled) particles in a mixed solution of 1 μm and 500 nm polystyrene particles at high laser intensity using opto-thermoelectric tweezers.
Supplementary Video 3: Real-time video showing the selective trapping of smaller 200-nm nanoparticles in a mixed solution of 200-nm (fluorescent-labeled) and 1-μm polystyrene particles at high average speckle intensity using opto-thermoelectric speckle tweezers.
Supplementary Video 4: Real-time video showing the large-scale filtration of 200-nm nanoparticles from a mixed solution of 200-nm (fluorescent-labeled) and 1-μm polystyrene particles flown in a microfluidic channel integrated with opto-thermoelectric speckle tweezers.
