# Supplementary material for: Opto-thermoelectric speckle tweezers
Source: Nanophotonics. Author manuscript; Available in PMC 2021 Jul 20. (PMC8291376; doi:10.1515/nanoph-2019-0530)
Supplement: supplementary notes and vides — Supplementary Video 1: Real-time video showing the trapping of both 500-nm (fluorescent-labeled) and 1-μm polystyrene particles at low laser intensity using opto-thermoelectric tweezers. Supplementary Video 2: Real-time video showing the selective trapping of 500-nm (fluorescent-labeled) particles in a mixed solution of 1 μm and 500 nm polystyrene particles at high laser intensity using opto-thermoelectric tweezers. Supplementary Video 3: Real-time video showing the selective trapping of smaller 200-nm nanoparticles in a mixed solution of 200-nm (fluorescent-labeled) and 1-μm polystyrene particles at high average speckle intensity using opto-thermoelectric speckle tweezers. Supplementary Video 4: Real-time video showing the large-scale filtration of 200-nm nanoparticles from a mixed solution of 200-nm (fluorescent-labeled) and 1-μm polystyrene particles flown in a microfluidic channel integrated with opto-thermoelectric speckle tweezers. [file NIHMS1625918-supplement-supplementary_notes_and_vides.zip › SI-Abhay.OTEST.Nanophotonics2020Feb_revised.docx]

**Supplementary Information**

Opto-Thermoelectric Speckle Tweezers

Abhay Kotnala, Pavana Siddhartha Kollipara and Yuebing Zheng*

Walker Department of Mechanical Engineering, Materials Science & Engineering Program and Texas Materials Institute, The University of Texas at Austin, Austin, TX 78712

*Corresponding author: [zheng@austin.utexas.edu](mailto:zheng@austin.utexas.edu)

1. Temperature distribution generated by the laser speckle field

We used a commercial finite element analysis solver (COMSOL Multiphysics V5.5) to simulate the temperature distribution generated by the laser speckle incident at the substrate/solution interface. A three-dimensional (3D) model made up of a box of 1mm ×1mm × 0.5 mm was used to obtain the temperature distribution within water. Predefined modules of ‘Heat transfer in fluids’ were used to calculate the temperature distribution. A heating source with a distribution similar to the speckle intensity distribution shown in Figure S1b was placed at the bottom interface to model the opto-thermal heating from the speckle beam. The speckle intensity distribution was calculated based on the optical image of the speckle field (Figure S1a) and total output power of the multi-mode (MM) fiber. Image thresholding was applied to the speckle intensity distribution to extract the hotspots in the optical speckle field (Figure S1b). A constant absorption factor of 0.2 to account for the light-to-heat conversion by AuNIs was used to obtain the heat input at the bottom interface of the 3D model. Room temperature was set at all other boundaries.


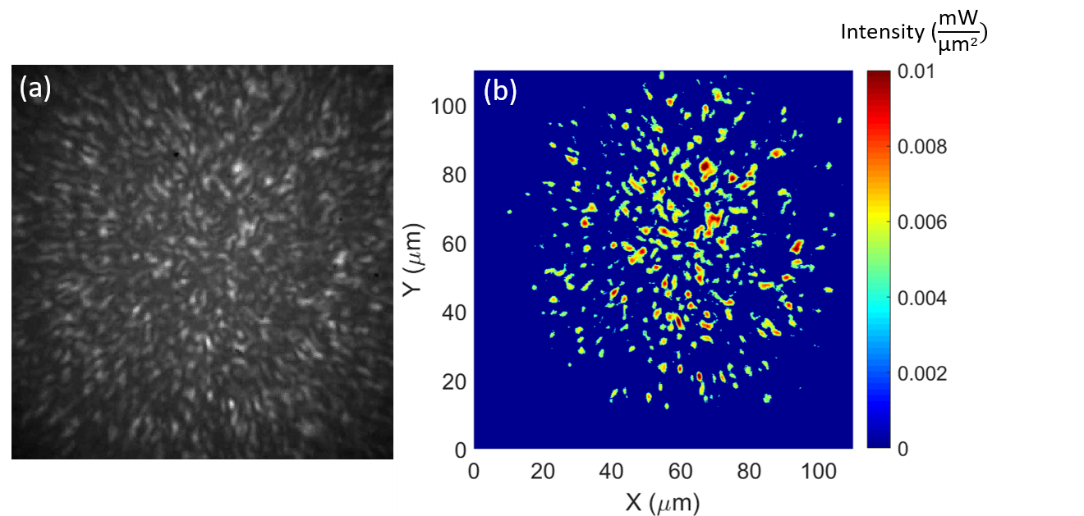


*Figure S1 (a) Optical image of a 532 nm laser speckle field generated at the output of a multimode fiber with a core diameter of 100 µm. (b) Intensity distribution of the hotspots in the speckle field extracted after image thresholding and calculated based on the total output power of the MM fiber.*

The simulated temperature distribution (XY plane) and the corresponding temperature gradient ($\nabla$T) generated from the speckle field are shown in Figures S2a and S2b, respectively. The thermal speckle field shows several hotspots with temperature reaching as high as 22 K above room temperature (293 K). The temperature of the hotspots present at the center of the speckle field were higher due to the heat diffusion from several surrounding thermal hotspots. The maximum temperature-gradient obtained for the thermal speckle field was 2.3 × 10^6^ K/m. Figure S2c shows the zoomed-in image of the temperature gradient distribution shown in the area marked by the box in Figure S2b. The arrows converging to the regions of near-zero temperature gradient, surrounded by the maximum temperature gradient, indicate the trapping locations in the thermal speckle field. The arrows in other zero temperature gradient regions pointing outwards correspond to an unstable equilibrium region from which the particles get repelled.


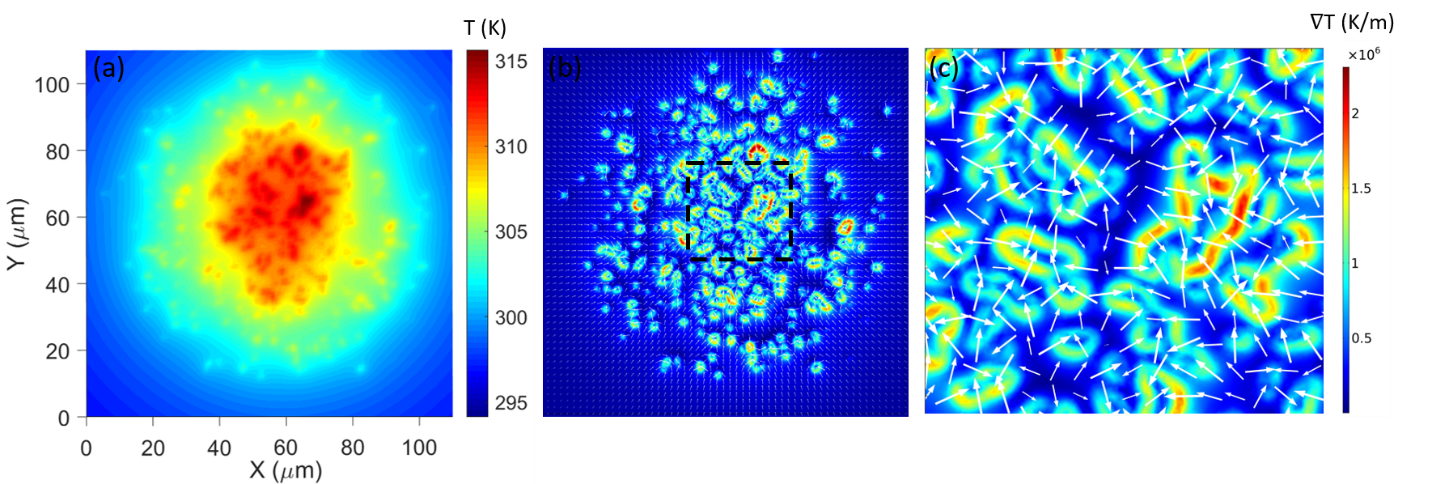


Figure S2 (a) Temperature distribution (XY plane) generated at the AuNI surface when excited with a speckle intensity pattern shown in Figure S1b. (b) Temperature gradient distribution obtained from Figure S2a. (c) Zoomed-in image of the temperature gradient distribution shown in the area marked by the box in Figure S2b. The arrows converging to the regions of near-zero temperature gradient surrounded by high temperature gradient areas represent the trapping locations in the thermal speckle field.


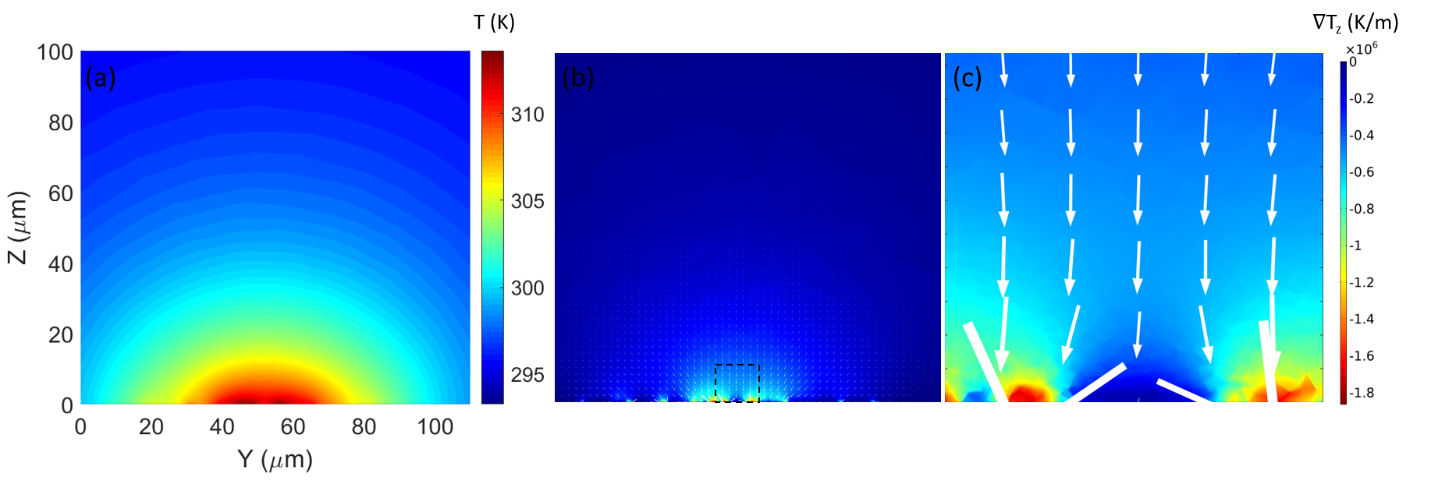
*Figure S3 (a) Temperature distribution (YZ plane) generated at the AuNI surface when excited with a speckle intensity pattern shown in Figure S1b. (b) Temperature gradient distribution obtained from Figure S3a. (c) Temperature gradient distribution of the area marked by the box in Figure S3b. The arrows point to the direction of trapping of particles.*

The temperature distribution normal to the substrate (YZ plane) and the temperature-gradient ( $\nabla T_{Z}$) were also calculated as shown in Figures S3a and S3b, respectively. Figure S3c shows the zoomed-in image of the temperature gradient distribution shown in the area marked by the box in Figure S3b, where the arrows point to the direction of trapping of particles.

1. Calculation of optical forces in opto-thermoelectric speckle tweezers (OTEST)

The optical forces acting on the polystyrene particles trapped at the hotspots of a speckle field were determined using the Maxwell Stress Tensor (MST) approach^1^. The time-averaged optical force acting on a particle due to the harmonic field can be found by integrating the MST over a closed surface surrounding the particle. The components of MST were calculated from the electric field distribution obtained using commercially available simulation software based on finite-difference time-domain method (FDTD solutions, Lumerical Inc). The refractive index of polystyrene particles as a function of wavelength was taken from the work of Sultanova et al.^2^ To simulate the electric field distribution, a Gaussian beam with intensity and beam-waist corresponding to the average maximum intensity and size (half-power beam width or HPBW) of the hotspots in the speckle field was used as an input source in the simulations. Based on the optical image of the speckle field (Figure S4a) and total output power of the MM fiber, we calculated the intensity distribution of the speckle field (Figure S4b). Individual hotspots (Figure S4c) selected from the speckle field were used to obtain the maximum intensity and size of the hotspots by fitting a Gaussian function to the intensity variation along the major-axis direction of a single speckle hotspot (Figure S4d).


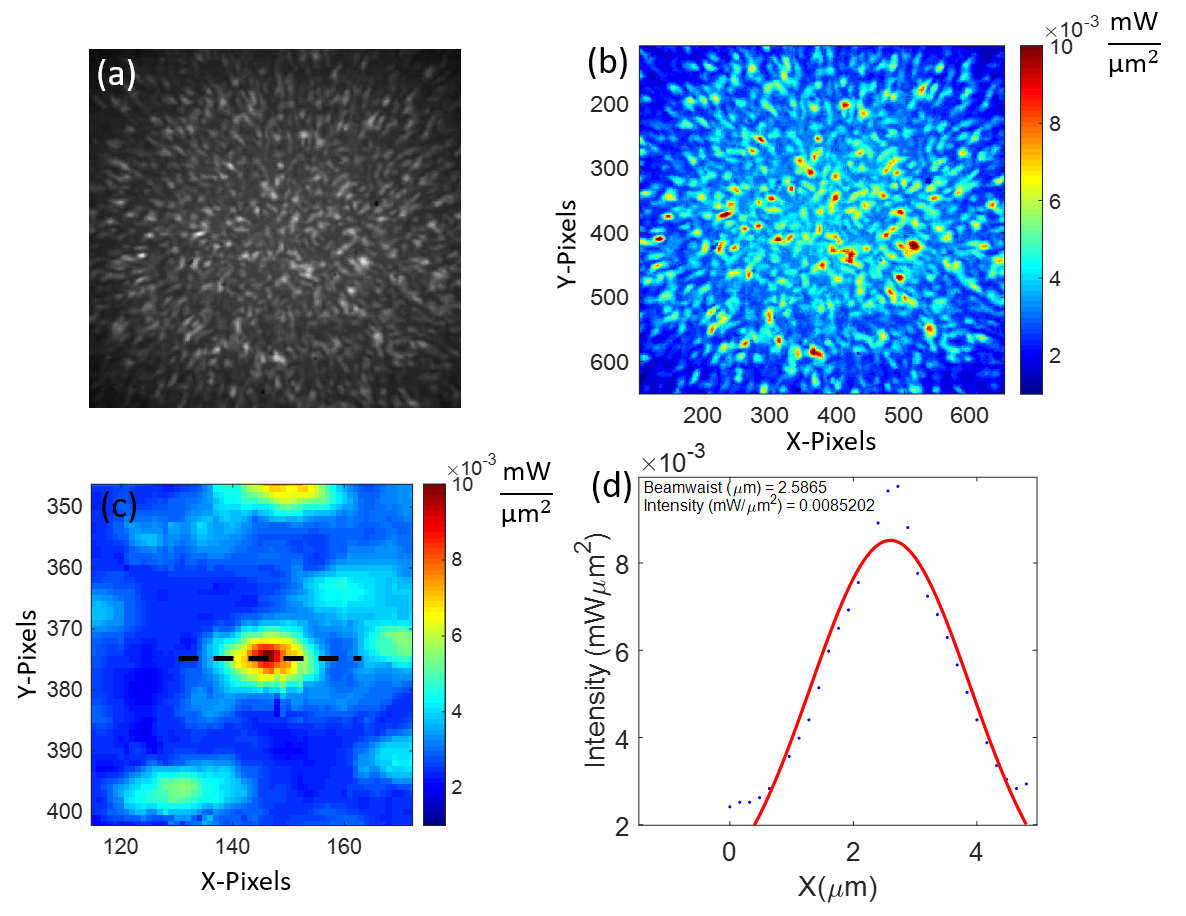


Figure S4 (a) Optical image of a 532 nm laser speckle pattern generated at the output of a multimode fiber with a core diameter of 100 µm. (b) Intensity map of the speckle field calculated based on the total output power of the MM fiber and the optical image shown in Figure S4a. Each pixel size equals 0.16 µm. (3) A zoomed-in image of a hotspot obtained from Figure S4b. (d) Intensity variation along the major-axis of the hotspot (dashed line) shown in Figure S4c. The red curve shows the Gaussian fit to the intensity variation.

The average maximum intensity and HPBW of the hotspots in the thermal speckle field was found to be 0.007± 0.001 mW/µm^2^ and 2.78± 0.35 µm, respectively (sampling number N=11). The in-plane and normal component of the total optical forces were calculated for 1 µm, 500 nm and 200 nm particles as shown in Figures S5a and S5b, respectively. The optical forces acting on the particles were in the sub-femtonewtons range, which were typically three orders of magnitude smaller than the thermoelectric forces acting on the particles for the laser intensities used in this work^3^.


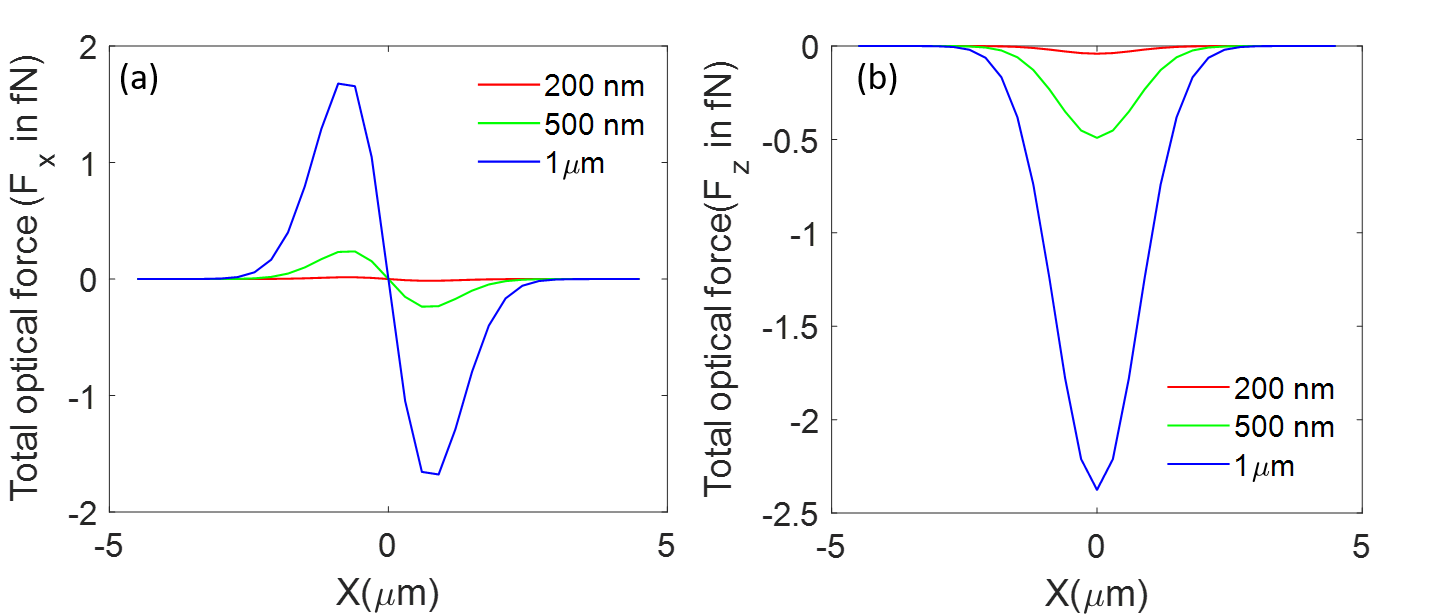


*Figure S5 (a) In-plane and (b) normal component of total optical force acting on 200 nm, 500 nm and 1 µm particles in an optical speckle field.*

1. Large-scale trapping of particles of different sizes and material compositions by OTEST

Figures S6a, S6b and S6c show the trapping of many 2 µm, 1 µm and 200 nm (fluorescent-labeled) polystyrene particles in a thermal speckle field, respectively. To enable thermoelectric forces, the particles were dispersed in 2mM CTAC solutions. An average speckle intensity of <I> = 1.6 μW/μm^2^ was used to trap 2 and 1 µm particles, while 4.2 μW/μm^2^ was used to trap the smaller 200 nm nanoparticles. Silver nanoparticles of a diameter of 200 nm dispersed in 10 mM CTAC solution were also trapped by OTEST at an average speckle intensity of <I> = 4.2 μW/μm^2^ (Figure S6d).


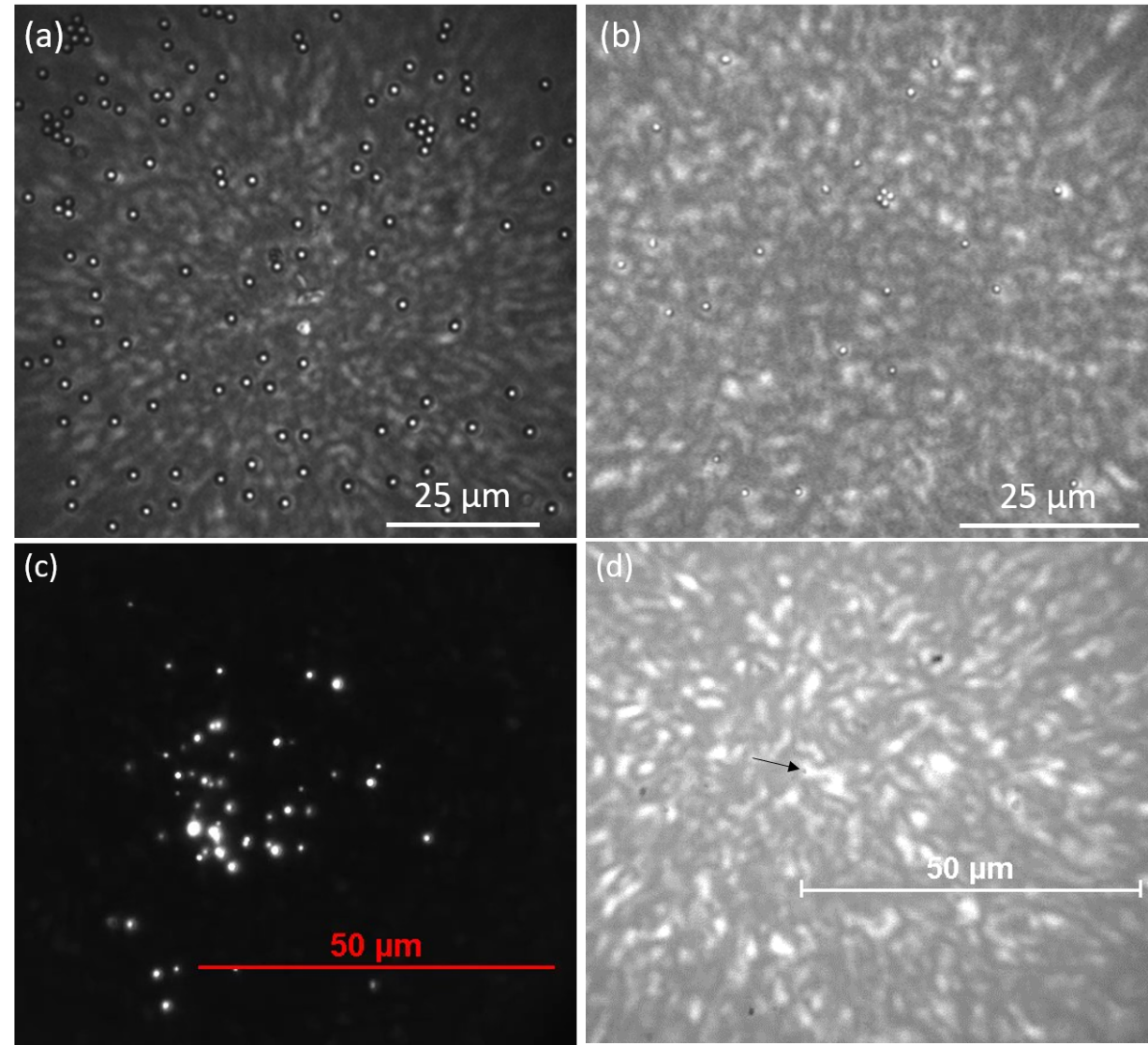


*Figure S6. Optical images showing trapping of multiple polystyrene particles of diameters of (a) 2 µm (b) 1 µm and (c) 200 nm (fluorescent-labelled) in a thermal speckle field. (d) Bright-field optical image showing trapping of multiple 200 nm silver nanoparticles by OTEST. The arrow points to one of the nanoparticles trapped in the speckle field.*

1. Selective trapping of particles by opto-thermoelectric tweezers (OTET)

The trapping behavior of particles in OTET is dependent on the incident laser intensity. Typically, at low incident powers of less than 1 mW, the AuNI substrate can generate a strong temperature gradient, which results in a strong thermoelectric force responsible for trapping of particles. By increasing the incident laser intensity, the thermoelectric force acting on the particle can be further increased as it increases both the temperature and the temperature gradient at the AuNI substrate. However, the rise in temperature also generates a localized convection flow, which imparts a drag force on the particle in a direction opposite to the thermoelectric force at the trapping location. Thus, at high laser intensities, the trapping of particles in OTET is governed by the balance between the two competing forces acting on the particle: the thermoelectric force and the drag force from localized convection flow. By modulating the incident laser intensity in OTET, the balance between the thermoelectric force and the drag force convection can be controlled to selectively trap particles based on their size .

A typical OTET set-up (Figure S7a) consisting of a single 532 nm laser beam focused on an AuNI substrate using a 50× objective lens was used to demonstrate selective trapping of the smaller 500 nm-diameter particles from a mixture of 500 nm and 1 µm particles. At low incident laser power of 0.9 mW, both 500 nm and 1 µm polystyrene particles were stably trapped by OTET as shown in Figures S7b and S7c (See Supplementary Video 1). However, when the incident laser power was increased to 1.6 mW, only the 500 nm particles could be trapped in the thermal hotspot created by the laser beam (Figure S7e), while the 1 µm particles, after getting initially pulled towards the hotspot, got dislodged from the trap and could not be trapped (Figure S7d) (See Supplementary Video 2). At the higher laser intensity, the drag force due to the localized convection flow increases significantly compared to the thermoelectric force acting on the 1 µm particle, which results in the displacement of the particle out of the trap. The displacement of the particle away from the surface of the AuNI substrate in the +Z direction at the trap location confirms the presence of a localized convection flow responsible for dislodging of the particle. It should be noted that, for the given OTET set-up, the optical scattering force acting on the particle is in the -Z direction, which pushes the particle towards the AuNI substrate and cannot be responsible for displacement of the particle out of the trap. On the other hand, due to the comparatively lower drag force on the 500 nm particles, the thermoelectric force remains dominant for the 500 nm particles, which can still be trapped by OTET. Thus, by controlling the laser power intensity, we can selectively trap particles of different sizes in OTET.


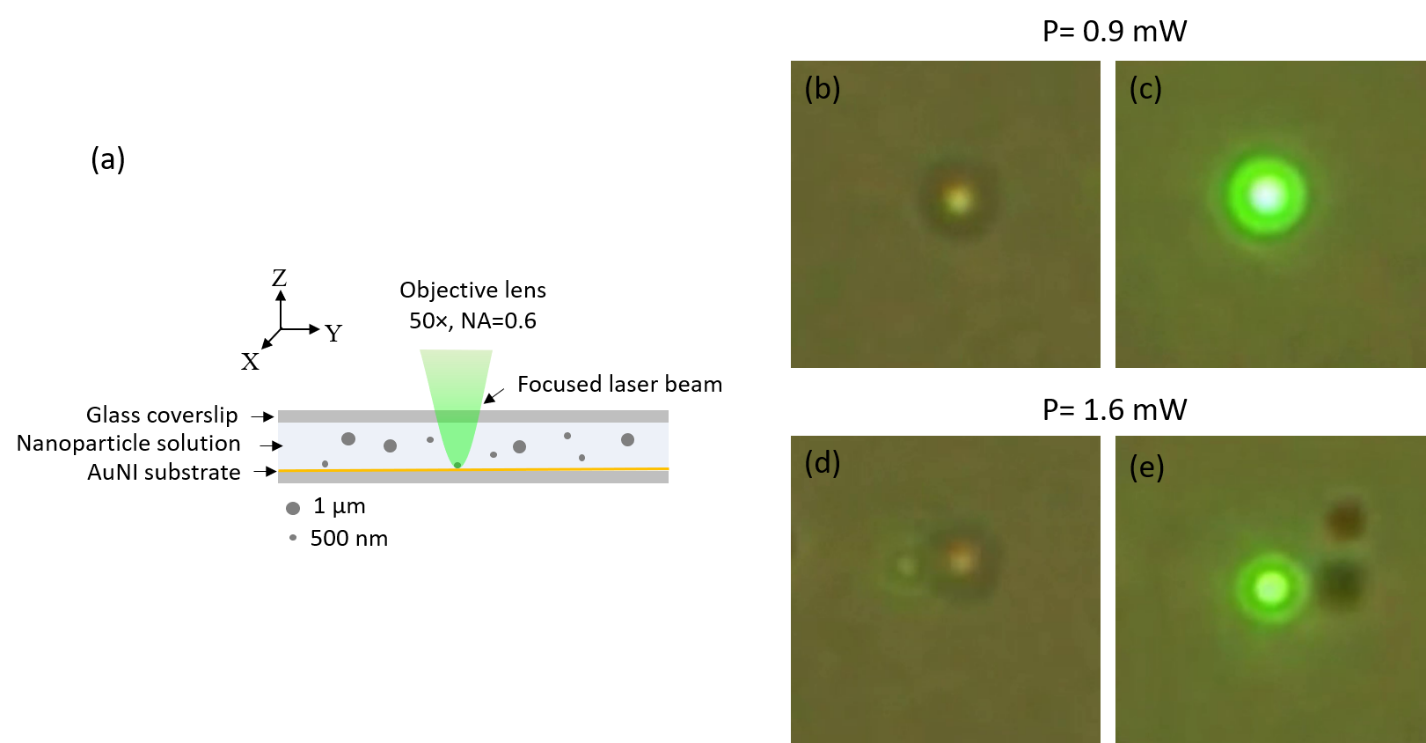


Figure S7 (a) Schematic of the experimental-setup for opto-thermoelectric trapping of 500 nm and 1 µm polystyrene particles using a single laser beam. (b & c) Optical images showing trapping of 1 µm (non-fluorescent) and 500 nm (fluorescent) polystyrene particle, respectively, at low laser power of 0.9 mW. (d) Optical image of a 1 µm particle dislodged from the trap at high laser power of 1.6 mW. (e) Optical image of a trapped 500 nm (fluorescent) polystyrene particle at high laser power of 1.6 mW. The non-fluorescent 1 µm particles in the image are the dislodged particle from the trap.

Similar to OTET, OTEST can be used to selectively trap a large number of particles in the thermal speckle field. In our demonstration, an optical set-up similar to that shown in Figure 1a was used for trapping of 200 nm and 1 µm polystyrene particles dispersed in a solution. The speckle field from the output of a MM optical fiber was incident on an AuNI substrate from the top as shown in Figure S8a. For this OTEST configuration, the optical scattering force and thermoelectric force acting on the particles are in the -Z direction similar to the direction of the speckle laser beam, which pushes the particles to the AuNI substrate. At low average speckle intensity of <I> = 1.4 μW/μm^2^, both 200 nm (fluorescent-labelled) and 1 µm (non-fluorescent) polystyrene particles were trapped in the thermal speckle field as shown in Figure S8b. However, when the average speckle intensity was increased to <I> = 3.2 μW/μm^2^, the larger 1 µm particles got displaced from the thermal hotspots, while the 200 nm particles remained trapped in the thermal speckle field as shown in Figure S8c (See Supplementary Video 3). The increase in the average speckle intensity creates a localized convection flow in the solution chamber, which introduces a drag force on the particles in the +Z direction at the trapping spots. The drag force is opposite to the dominant thermoelectric forces (-Z direction) and pushes the particle out of the trap. The larger drag force compared to the thermoelectric force on 1 µm particle results in the dislodging of the particles from the speckle hotspots. However, due to the comparatively lower drag force on the smaller 200 nm particles with respect to the 1 µm particles, the smaller particles still remain trapped in the speckle field, leading to the selective trapping of particles based on their sizes by OTEST.


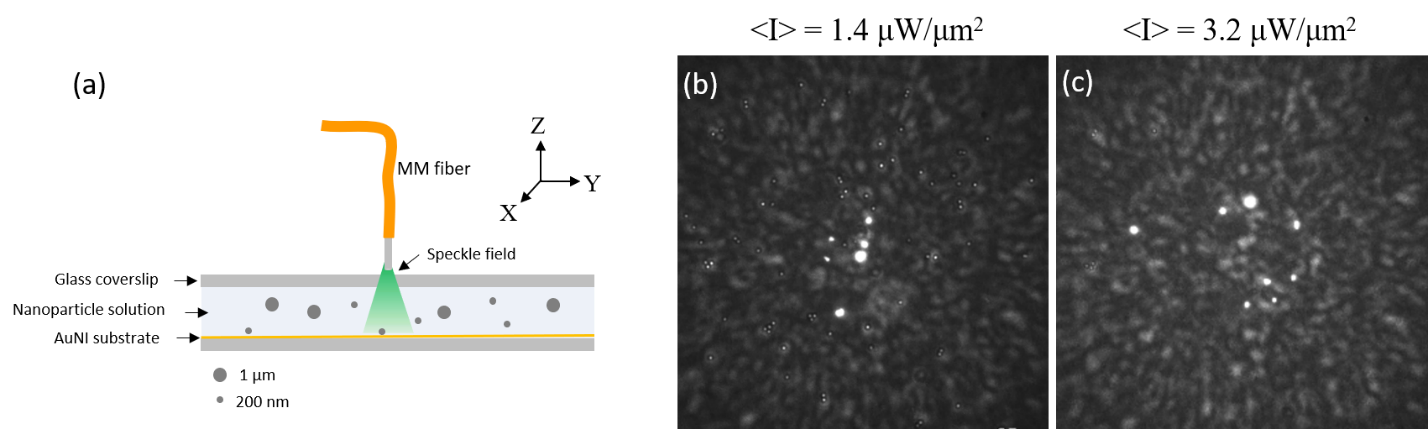


Figure S8 (a) Schematic of the experimental set-up used for trapping of 200 nm (fluorescent-labelled) and 1 µm (non-fluorescent) polystyrene particles using OTEST. (b) Optical image showing trapping of many 1 µm and 200 nm particles in a thermal speckle field at an average speckle intensity of <I> = 1.4 μW/μm^2^. (c) Optical image showing trapping of only 200 nm polystyrene particles in the thermal speckle field while most of the trapped 1 µm particles (Figure S8a) get dislodged from the thermal hotspots at an average speckle intensity of <I> = 3.2 μW/μm^2^.

1. Fabrication of plasmonic substrates integrated with microfluidic channels

Figure S9a shows the stepwise process for fabricating microfluidic channels on a AuNI plasmonic substrate. Figure S9b shows an optical image of a section of an AuNI-embedded microfluidic channel, which supports a thermal speckle field and microfluidic flow for size-based filtration of nanoparticles. The width and thickness of the microfluidic channel were 300 µm and 5 µm, respectively.


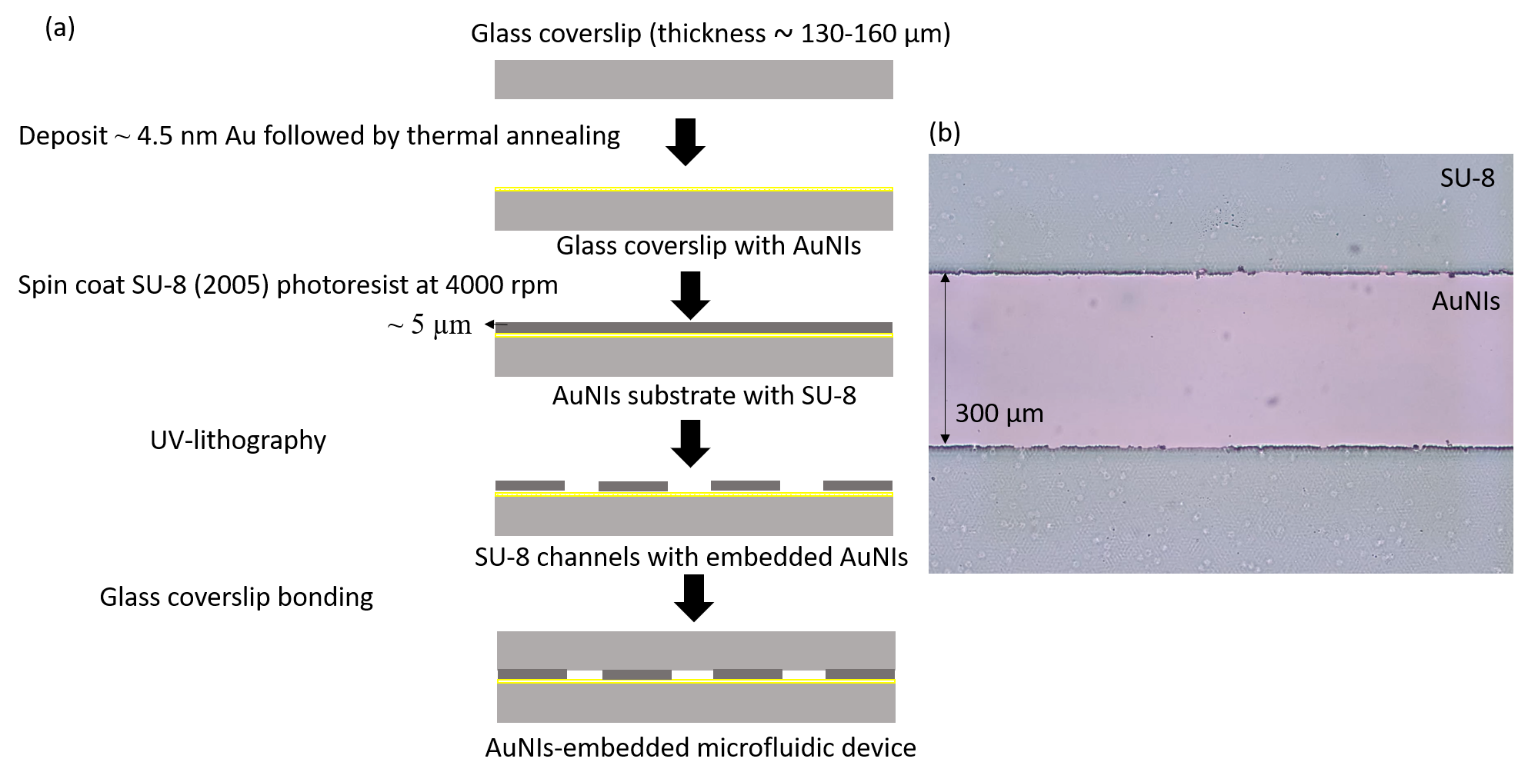


Figure S9 (a) Schematic of the fabrication process of a AuNI plasmonic substrate integrated with a microfluidic system that leads to an array of AuNI-embedded microfluidic channels. (b) Optical image of a section of an AuNI-embedded microfluidic channel.

References

1. Ye Q, Lin H. On deriving the Maxwell stress tensor method for calculating the optical force and torque on an object in harmonic electromagnetic fields. *Eur J Phys*. 2017;38:45202.

2. Sultanova NG, Nikolov ID, Ivanov CD. Measuring the refractometric characteristics of optical plastics. *Opt Quantum Electron*. 2003;35:21-34.

3. Kollipara PS, Lin L, Zheng Y. Thermo-Electro-Mechanics at Individual Particles in Complex Colloidal Systems. *J Phys Chem C*. 2019;123:21639-21644.
